# A comparative study of mechanical strain, icariin and combination stimulations on improving osteoinductive potential via NF-kappaB activation in osteoblast-like cells

**DOI:** 10.1186/s12938-015-0039-z

**Published:** 2015-05-21

**Authors:** Qiang-Song Wang, Xin-Chang Zhang, Rui-Xin Li, Jing-Gong Sun, Wei-Hua Su, Yong Guo, Hao Li, Xi-Zheng Zhang

**Affiliations:** Tianjin Institute of Medical Equipment, Academy of Military Medical Sciences, No. 106 Wandong Road, Hedong District, Tianjin, 300162 People’s Republic of China

**Keywords:** Mechanical strain, Icariin, BMP-2, NF-κB, Bone remodeling

## Abstract

**Background:**

The combination of drugs and exercise was the effective treatment in bone injure and rebuilding in clinic. As mechanical strain has potential in inducing the differentiation of osteoblasts in our previous study, the further research to investigate the combination of mechanical strain and icariin stimulation on inducing osteoblast proliferation, differentiation and the possible mechanism in MC3T3-E1 cell line.

**Methods:**

A whole cell enzyme-linked immunosorbent assay that detects the bromodeoxyuridine incorporation during DNA synthesis was applied to evaluate the proliferation. The mRNA expression of alkaline phosphatase (ALP), osteocalcin (OCN), type I collagen (Col I), bone morphogenetic protein-2 (BMP-2) and BMP-4 was detected by real-time reverse-transcription polymerase chain reaction. The activity of ALP was analyzed by ELISA and the protein expression of OCN, Col I and BMP-2 was assessed by western blot. Moreover, the activity of nuclear transcription factor kappa-B (NF-κB) signaling pathway was investigated with the expression of inhibitor of κB (IκB) α, phosphorylation of IκB-α (P-IκB-α), p65, P-p65 by western blot.

**Results:**

We observed that compared to single mechanical strain or icariin stimulation, the mRNA and protein expressions of ALP (*P* < 0.05 or *P* < 0.01), OCN (*P* < 0.01) and Col I (*P* < 0.05 or *P* < 0.01) were increased significantly by the combination of mechanical strain and icariin stimulation. Moreover, the combination of mechanical strain and icariin stimulation could up-regulate the expression of BMP-2 (*P* < 0.01) and BMP-4 compared to single mechanical strain or icariin stimulation. The combination of mechanical strain and icariin stimulation could activate NF-κB signaling pathway by increasing the expression of IκB α, P-IκB-α, p65, P-p65 (*P* < 0.01).

**Conclusion:**

The combination of mechanical strain and icariin stimulation could activate the NF-κB pathway to improve the proliferation, differentiation of osteoblast-like cells.

## Background

The regular load is important for maintaining the integrity of bone described by Wolff’s law on the relationship between bone morphology and mechanical load [1]. Disuse or a lack of load, such as prolonged bed rest, spinal cord injury or space flight, could result in the rapid loss of bone mass and even osteoporosis in some cases [2]. Moreover, pathological bone modeling, remodeling, or microdamage resulted from accumulated overloaded strain could result in fracture of bone [3–5].

Bone remodeling is a complex biological process depending on genetic, hormonal, metabolic and mechanical environment [6]. Mechanical loading plays an important role in the regulation of bone remodeling, since suitable mechanical stimulation could maintain normal bone volume [7]. Mechanical stimulation could be transmitted through the extracellular matrix (ECM) to resident osteoblasts, osteocytes, periosteal cells and osteoclasts in bone [8]. Osteoblasts were important mechanical receptors that transformed mechanical stimulation into biochemical signals, and also could secrete bone matrix to promote bone matrix mineralization [9].

Mechanical strain could not only promote matrix mineralization of osteoblasts [10], but increase the expression of ECM-related proteins of osteoblasts, such as bone morphogenetic protein 2 (BMP-2), osteopontin (OPN), osteocalcin (OCN) and type I collagen (Col I) [11]. Bone morphogenetic proteins (BMPs), a kind of secretory glycoprotein purified from bone matrix, are potent osteoblastic differentiation factors in remodeling and formation of bone [12]. Our previous study showed that mechanical strain can activate p38 mitogen-activated protein kinase (MAPK)/NF-κB pathway by up-regulating BMP-2 expression in MC3T3-E1 cells [13]. Biomechanical signals are essential for bone homeostasis, growth, adaptation, healing and remodeling [14–17]. Mechanical strains have been shown to activate many types of signal transduction cascades, including the NF-κB signal pathway [18–20]. NF-κB exists in most cells as homodimeric or heterodimeric complexes of p50 and p65 subunits and remains inactive in the cytoplasm of cells associated with the NF-κB inhibitory protein (I-κB) [21]. Activated NF-κB could increase nuclear p65 protein associated with decreased cytosolic I-κB protein [21]. The resulting free NF-κB is then translocated into the nucleus, where it binds to κB binding sites in the promoter region of target genes [22–24]. Our previous study has shown that mechanical strain could induce osteoblast proliferation, differentiation and mineralization by activating NF-κB pathway [13]. Although mechanical loading plays an important role in the regulation of bone remodeling, drugs are still the first choice for treatment of bone injury and remodeling.

Icariin, the main active flavonoid glucoside isolated from Herba epimedii (HEF), is used in traditional Chinese medicine for treatment of kidney, joints, and other disorders. It was shown that icarrin could prevent ovariectomy-induced bone loss and restored femoral strength [25–27]. Moreover, icariin could induce osteoblast proliferation, differentiation and mineralization though estrogen receptor-mediated extracellular signal-regulated kinases (ERK) and c-Jun N-terminal kinase (JNK) signal activation [28]. It was reported by National Aeronautics and Space Administration (NASA)’s Johnson Space Center that exercise with medicine (alendronate pill and vitamin D) could prevent the loss of bones and muscle mass of astronauts compared to those using exercise or drug only [29]. Since exercise or drug alone has not been entirely successful in accelerating bone remodeling and preventing bone loss, the combined effects of exercise and drugs may be a better choice. Although our previous studies have shown that mechanical strain could induce osteoblast proliferation, differentiation and mineralization, the combination of mechanical strain and icariin stimulation was not investigated, moreover, the mechanism of the combination of mechanical strain and icariin on inducing osteoblast proliferation, differentiation was still not clear.

The present study was aimed to study the mechanical strain, icariin and the combination of mechanical strain and icariin stimulation on inducing osteoblast proliferation, differentiation, and investigate the possible mechanism in osteoblast-like cells (MC3T3-E1), which will provide strong scientific evidence for the treatment of bone injury and remodeling by combination of exercise and drugs in clinic.

## Methods

### Reagents

The α-modified Eagle’s medium–high glucose (α-MEM) was purchased from Sigma-Aldrich Co. (St. Louis, MO, USA). Cell Proliferation ELISA (BrdU) kit was purchased from Roche (Mannheim, Germany). ALP vitality assay kit was purchased from Nanjing Jiancheng Biotechnology Co., Ltd. (Jiangsu, China). BCA Protein Assay Kit was obtained from Pierce (Rockford, IL, USA). Mammalian Cell Lysis Kit and UNIQ-10 column Trizol total RNA extraction kit were bought from Sangon Biological Engineering Technology and Services Co., Ltd. (Shanghai, China). Improm-II Reverse Transcription System was purchased from Promega Corporation (Madison, WI, USA). FastStart Universal SYBR Green Master (ROX) kit was purchased from Roche (Mannheim, Germany). P-IκB-α, IκB-α, p65, P-p65 monoclonal antibodies and peroxidase-conjugated secondary antibody were purchased from Cell Singaling Technology (USA), and β-actin monoclonal antibody was purchased from Sigma-Aldrich Co. (USA). NF-κB inhibitor BAY 11-7082 was purchased from Beyotime Institute of Biotechnology (China). Col I, OCN, BMP-2 monoclonal antibodies and peroxidase-conjugated secondary antibody were purchased from Santa Cruz Biotechnology (CA, USA). Icariin was purchased from the Chinese National Institute for Control of Pharmaceutical and Biological Products (Beijing, China).

### Cells and cell culture

Murine MC3T3-E1 cell line was obtained from Cell Culture Center of Chinese Academy of Medical Sciences (Beijing, China). MC3T3-E1 cells were maintained in α-MEM supplemented with 10% fetal bovine serum (FBS), 100 U/mL penicillin and 100 μg/mL streptomycin at 37°C in a humidified incubator containing 5% CO_2_. Icariin (1 × 10^−5^ M) was added in α-MEM 1 h prior to mechanical strain application and remained in the culture media throughout the experiment.

### Application of mechanical strain to cultured cells

**Figure 1:**
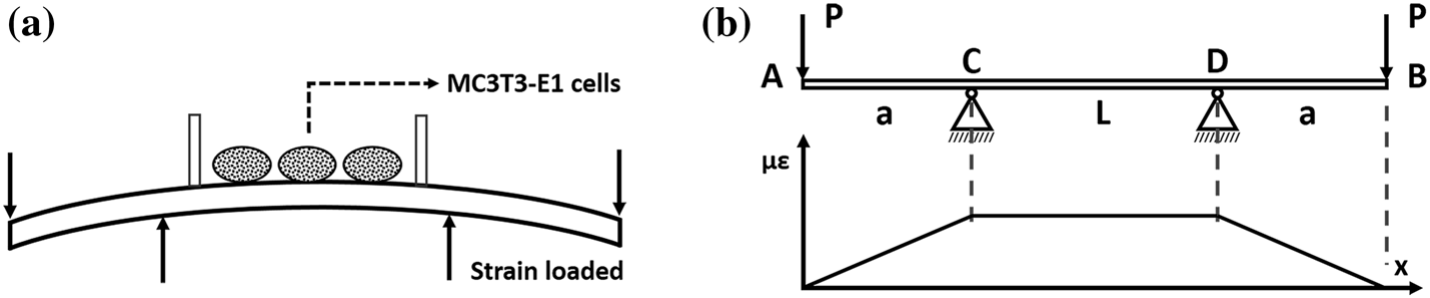
The loading device drawing (**a**) on MC3T3-E1 cells and strain distribution drawing of loading device (**b**). The cells seeded in *C*–*D* would be loaded with the same deformations.

The MC3T3-E1 cells were seeded on mechanical loading dishes that were reformed from cell culture dishes (Nalge Nunc International, Roskilde, Denmark) and subjected to mechanical tensile strain of 2,500 microstrain (με) at 0.5 Hz of 1 h/day for 3 days. The mechanical strain was generated by a specially designed four-point bending device, as previously described [30–32]. Control cells were incubated under the same conditions except for application of mechanical stimulation. The device was driven by a stepping motor (controlled by a single chip microcomputer) and has been shown to produce homogenous cell culture substrate that is composed predominantly of uniaxial cells with the same deformations [33, 34] (Figure 1).

### BrdU ELISA for cell proliferation

The proliferation of MC3T3-E1 cells were determined using a colorimetric immunoassay for the quantification of cell proliferation based on the detection of 5-bromo-20-deoxyuridine (BrdU) incorporation during DNA synthesis [35]. After stimulation of mechanical strain and icariin for 3 days, BrdU was added to a final concentration of 10 μM. After incubation for 18 h, DNA synthesis was assayed with the Cell Proliferation ELISA (BrdU) Kit (Roche, Germany) using colorimetric detection according to the manufacturer’s instructions. Newly synthesized BrdU-DNA was determined using a multifunctional microplate reader (FlexStation 3, Molecular Devices, USA).

### ALP activity analysis

After stimulation of mechanical strain and icariin, the cells were washed three times with cold PBS. Cells were collected by centrifugation and washed once with PBS. The washed cell pellets were resuspended in extraction lysis buffer (50 mM HEPES pH 7.0, 250 mM NaCl, 5 mM EDTA, 0.1% Nonidet P-40, 1 mM PMSF, 0.5 mM DTT, 5 mM NaF and 0.5 mM sodium orthovanadate) containing aprotinin and leupeptin (5 mg/mL), respectively, and incubated for 20 min at 4°C. Cell debris was removed by centrifugation, and supernatants were rapidly frozen. The protein was detected by BCA method (Pierce, USA). ALP activities in each sample were examined by an ALP vitality assay detection kit (Nanjing Jiancheng Biotechnology Co. Ltd, Nanjing, China) and the absorbance was measured at 520 nm on a multifunctional microplate reader (FlexStation 3, Molecular Devices, USA). Values of ALP activity were normalized by protein concentration.

### Real-time RT-PCR for detecting the mRNA expression of ALP, BMP-2, BMP-4, OCN, Col I

Total RNA was isolated using Sangon UNIQ-10 column Trizol total RNA extraction kit according to the instructions of the manufacturer. RNA (1 μg) was reversely transcribed using ImProm-II Reverse Transcription System cDNA synthesis kit. The real-time RT-PCR oligonucleotide primers used for mouse ALP, BMP-2, BMP-4, OCN, Col I and β-actin are shown in Table 1. The reactions were setup in duplicates in 25 μL total volumes with 1 μL of each primer (0.3 μM final concentrations), 12.5 μL of FastStart Universal SYBR Green Master (ROX) (Roche), and 1 μL of template. The PCR cycle was as follows: 95°C for 10 min, 40 cycles of 95°C for 15 s, 60°C for 1 min. A melt curve analysis was performed to verify that a single product per primer pair was amplified at the end of each experiment. The amplification and analysis were performed using an ABI Prism 7500 Real-Time PCR System. Samples were compared using the relative CT method. The fold decrease or increase was determined relative to a blank control after normalized to a housekeeping gene using $2^{{ - \rm{\Delta} \rm{\Delta} C_{\text{T}} }}$ [36, 37].

**Table 1:** The real-time RT-PCR oligonucleotide primers

Gene	Primer	Sequence (5′–3′)	PCR product (bp)
ALP	Forward	TGACCTTCTCTCCTCCATCC	183
	Reverse	CTTCCTGGGAGTCTCATCCT	
OCN	Forward	TGCTTGTGACGAGCTATCAG	148
	Reverse	GAGGACAGGGAGGATCAAGT	
Col I	Forward	GAGCGGAGAGTACTGGATCG	142
	Reverse	GTTCGGGCTGATGTACCAGT	
BMP-2	Forward	CCCCAAGACACAGTTCCCTA	169
	Reverse	GAGACCGCAGTCCGTCTAAG	
BMP-4	Forward	TGAGCCTTTCCAGCAAGTTT	180
	Reverse	CTTCCCGGTCTCAGGTATCA	
β-Actin	Forward	AGAGGGAAATCGTGCGTGAC	138
	Reverse	CAATAGTGATGACCTGGCCGT	

### Western blot for analyzing the protein expression of BMP-2, OCN, Col I

The cells were washed three times with cold PBS and collected by centrifugation and washed once with PBS. The washed cell pellets were resuspended in extraction lysis buffer (50 mM HEPES pH 7.0, 250 mM NaCl, 5 mM EDTA, 0.1% Nonidet P-40, 1 mM PMSF, 0.5 mM DTT, 5 mM NaF and 0.5 mM sodium orthovanadate) containing aprotinin and leupeptin (5 mg/mL), and incubated for 20 min at 4°C. Cell debris was removed by centrifugation, and supernatants were rapidly frozen. The protein was detected by BCA method (Pierce, USA). Of cellular protein, 40 mg protein from cell extracts was electro-blotted onto a polyvinylidene difluoride (PVDF) membrane following separation on a 10% SDS-polyacrylamide gel electrophoresis. The immunoblot was incubated for 1 h with blocking solution (5% skim milk) at room temperature, and then incubated overnight with a 1:500 dilution of anti-BMP-2, anti-OCN, anti-Col I, β-actin antibody (Cell Singaling Technology, USA) at 4°C. Blots were washed three times with Tween 20/Tris-buffered saline (TTBS) and then incubated with a 1:2,000 dilution of horseradish peroxidase-conjugated secondary antibody (Santa Cruz Biotechnology, USA) for 1 h at room temperature. Blots were washed five times with TTBS and then developed by Horseradish peroxidase substrate (Millipore Corporation, USA) and data were captured by exposure to Kodak BioMax Light films.

### NF-κB activity assay

Our previous study showed that NF-κB signaling pathway could be activated at 30 min and the maximum expression levels were observed at 60 min after initiating mechanical stimulation [13]. The cells were pre-treated with icariin (1 × 10^−5^ M) for 1 h, and then the cells were subjected to mechanical tensile strain of 2,500 με at 0.5 Hz for 1 h to investigate the combined effect of mechanical strain and icariin on the NF-κB activity. The expressions of IκB-α, P-IκB-α, p65, P-p65 were analyzed by western blot. The extraction and detection of total protein were performed as described above. Protein from treated and untreated cell extracts were electro-blotted onto a PVDF membrane following separation on a 10% SDS-polyacrylamide gel electrophoresis. The immunoblot was incubated for 1 h with blocking solution (5% skim milk) at room temperature, and then incubated overnight with a 1:1,000 dilution of anti-IκB-α, anti-P-IκB-α, anti-p65, anti-P-p65, β-actin antibody (Cell Singaling Technology, USA) at 4°C. Blots were washed three times with Tween 20/Tris-buffered saline (TTBS) and then incubated with a 1:5,000 dilution of horseradish peroxidase-conjugated secondary antibody (Cell Singaling Technology, USA) for 1 h at room temperature. Blots were washed five times with TTBS and then developed by Horseradish peroxidase substrate (Millipore Corporation, USA) and data were captured by exposure to Kodak BioMax Light films.

### Statistical analysis

All values were represented as mean ± SD from at least three independent experiments. Statistically significant differences between groups were determined by one way analysis of variance (ANOVA) followed by Scheffe’s multiple range test. The criterion for statistical significance was *P* < 0.01 or *P* < 0.05.

## Results

### Cell proliferation

**Figure 2:**
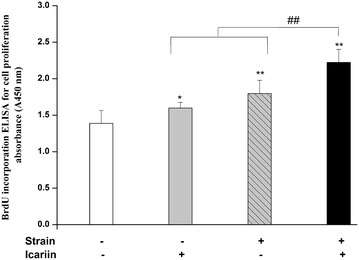
Effect of mechanical strain, icariin, the combination of mechanical strain and icariin stimulation on proliferation of MC3T3-E1 cells. After stimulation of mechanical strain, icariin, the combination of mechanical strain and icariin on MC3T3-E1 cells of 1 h/day for 3 days, BrdU was added and cells were reincubated for additional 18 h. BrdU incorporation was determined using a cell ELISA method. Data represent mean ± SD values from three independent experiments. *P < 0.05, **P < 0.01 (n = 4) compared with control group, ^#^ *P* < 0.05, ^##^ *P* < 0.01 compared with mechanical strain or icariin stimulation.

The comparable results for differences in proliferation behavior, expressed as the amount of newly synthesized DNA were shown in Figure 2. The DNA synthesis of MC3T3-E1 cells after mechanical strain, icariin, the combination of mechanical strain and icariin stimulation for 18 h revealed that MC3T3-E1 cells could proliferate after different stimulation. Compared to blank control group, proliferation with mechanical strain, icariin, the combination of mechanical strain and icariin stimulation was significantly higher (*P* < 0.01 or *P* < 0.05) than blank control group. In addition, the cell proliferation with the combination of mechanical strain and icariin stimulation was significantly higher than that of mechanical strain or icariin alone (*P* < 0.01).

### Effect of mechanical strain, icariin, combination of mechanical strain and icariin stimulation on ALP activity in MC3T3-E1 cells

**Figure 3:**
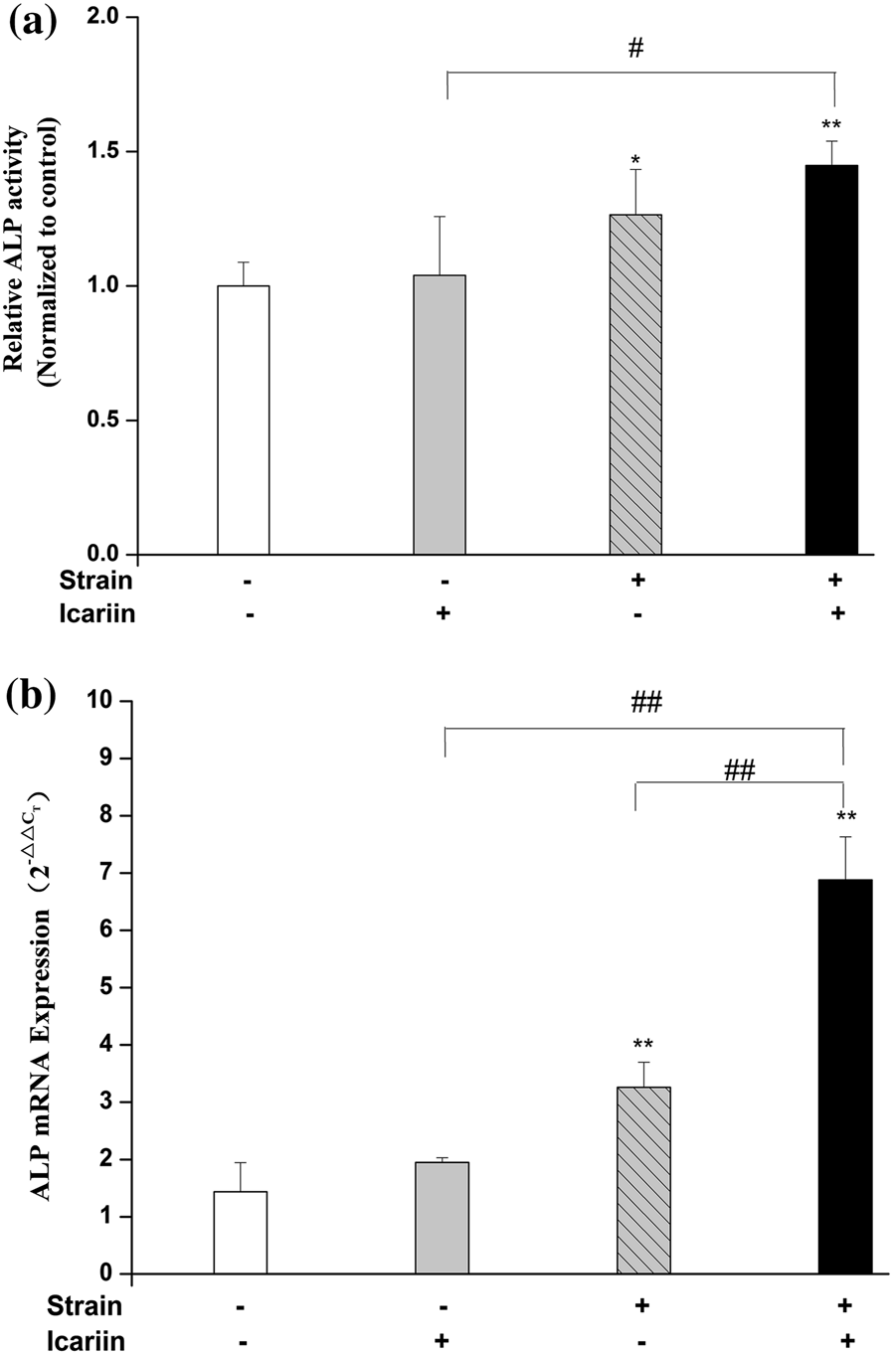
Effect of mechanical strain, icariin, the combination of mechanical strain and icariin stimulation on ALP activity and mRNA expression. After stimulation of mechanical strain, icariin, the combination of mechanical strain and icariin on MC3T3-E1 cells of 1 h/day for 3 days, the total protein and mRNA of MC3T3-E1 cells were extracted. The ALP activity was analyzed by ALP vitality assay detection kit (**a**) and the mRNA expression of ALP was analyzed by real-time RT-PCR (**b**). Data represent mean ± SD values from three independent experiments. *P < 0.05, **P < 0.01 (n = 4) compared with control group, ^#^ *P* < 0.05, ^##^ *P* < 0.01 compared with mechanical strain or icariin stimulation.

ALP was believed to be one major characteristics of osteoblast activity. As shown in Figure 3a, ALP activity was increased significantly (*P* < 0.01 or *P* < 0.05) after mechanical strain, the combination of mechanical strain and icariin stimulation compared to blank control group. Furthermore, compared to the stimulation of icariin, the combination of mechanical strain and icariin stimulation could increase ALP activity remarkably (*P* < 0.05). The mRNA expression of ALP was also investigated after mechanical strain, icariin, combination of mechanical strain and icariin stimulation in MC3T3-E1 cells. As shown in Figure 3b, the mRNA expression of ALP was up-regulated significantly (*P* < 0.01) after mechanical strain, the combination of mechanical strain and icariin stimulation compared to blank control group. Moreover, the increased level of combination of mechanical strain and icariin stimulation was notable than the stimulation of mechanical strain.

### Effect of mechanical strain, icariin, combination of mechanical strain and icariin stimulation on BMP-2, BMP-4 expression

**Figure 4:**
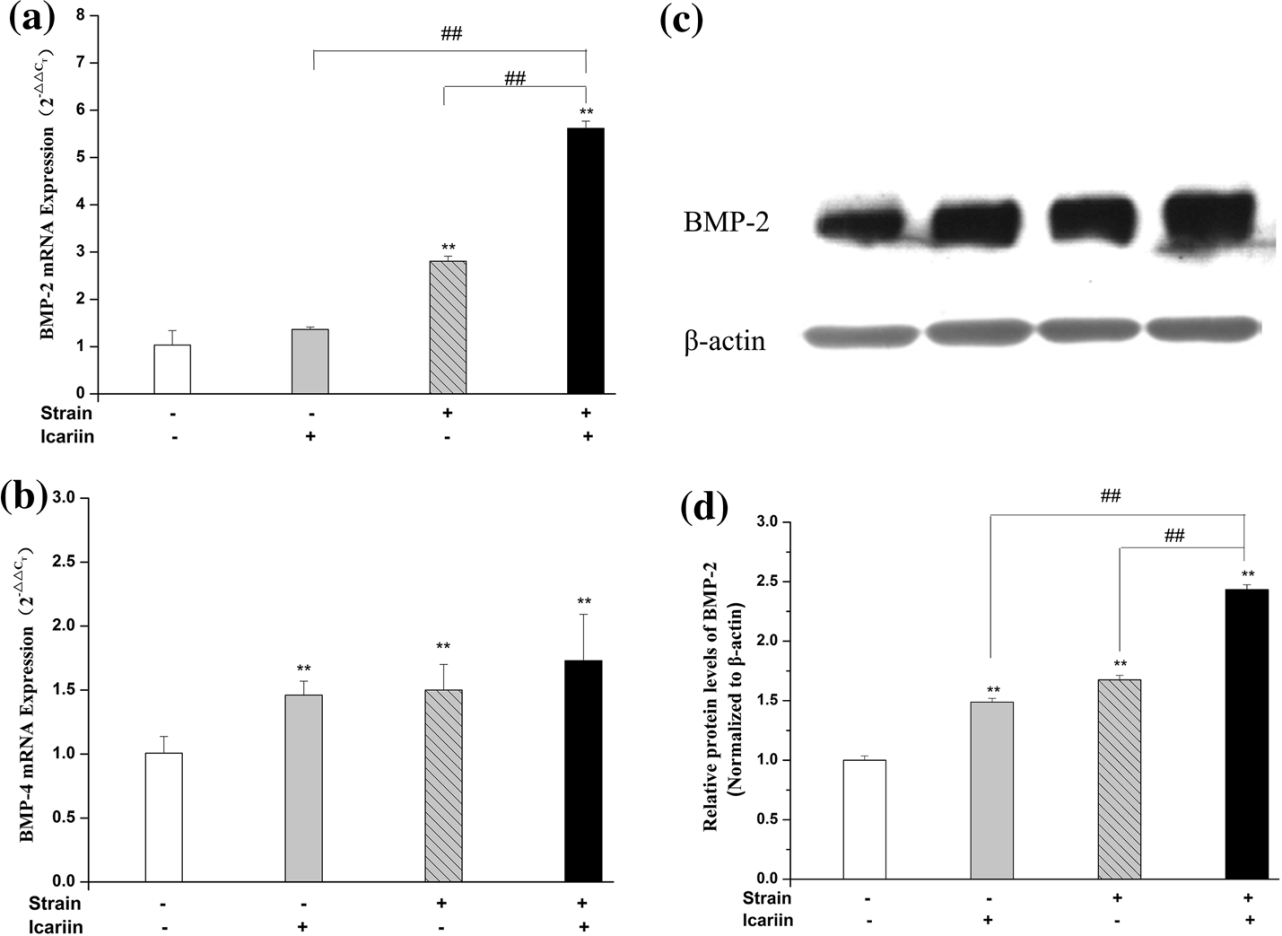
Effect of mechanical strain, icariin, the combination of mechanical strain and icariin stimulation on expression of BMP-2 and BMP-4. After stimulation of mechanical strain, icariin, the combination of mechanical strain and icariin on MC3T3-E1 cells of 1 h/day for 3 days, the total protein and mRNA of MC3T3-E1 cells were extracted. The mRNA expression of BMP-2 (**a**), BMP-4 (**b**) was analyzed by real-time RT-PCR, and the protein expression of BMP-2 (**c**) was detected by western blot. The β-actin protein was used as internal controls. Optical density analysis (**d**) was obtained with the Image-Pro Plus version 6.0. Data represent mean ± SD values from three independent experiments. *P < 0.05, **P < 0.01 (n = 4) compared with control group, ^#^ *P* < 0.05, ^##^ *P* < 0.01 compared with mechanical strain or icariin stimulation.

The mRNA expressions of BMP-2 and BMP-4 were investigated using real-time RT-PCR. As shown in Figure 4, compared to the blank control group, the mRNA expression of BMP-2 was up-regulated significantly (*P* < 0.01) after mechanical strain, the combination of mechanical strain and icariin stimulation in MC3T3-E1 cells. The combination of mechanical strain and icariin stimulation had significant advantages on up-regulating the mRNA expression of BMP-2 than the stimulation of mechanical strain or icariin alone (*P* < 0.01). Moreover, the protein expression of BMP-2 was investigated by western blot. As shown in Figure 4, the combination of mechanical strain and icariin stimulation could increase the protein expression of BMP-2 remarkably (*P* < 0.01) compared to the stimulation of mechanical strain or icariin alone. The mRNA expression of BMP-4 was up-regulated significantly (*P* < 0.01) after mechanical strain, icariin, the combination of mechanical strain and icariin stimulation compared to the blank control group in MC3T3-E1 cells.

### Effect of mechanical strain, icariin, combination of mechanical strain and icariin stimulation on OCN, Col I expression

**Figure 5:**
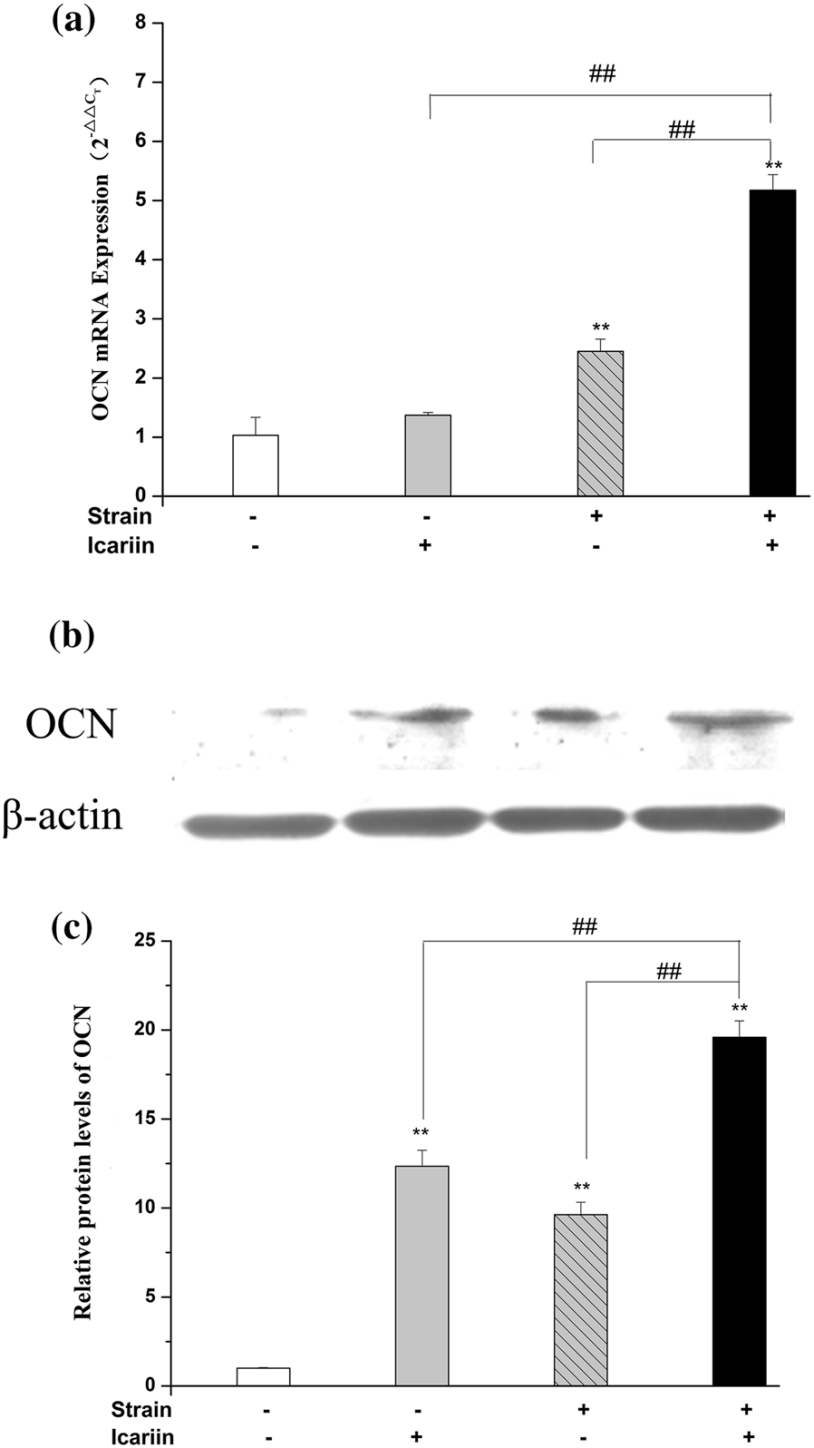
Effect of mechanical strain, icariin, the combination of mechanical strain and icariin stimulation on the protein and mRNA expression of OCN. After stimulation of mechanical strain, icariin, the combination of mechanical strain and icariin on MC3T3-E1 cells of 1 h/day for 3 days, the total protein and mRNA of MC3T3-E1 cells were extracted. The mRNA expression of OCN was analyzed by real-time RT-PCR (**a**), and the protein expression of OCN (**b**) was detected by western blot. The β-actin protein was used as internal control. Optical density analysis (**c**) was obtained with the Image-Pro Plus version 6.0. Data represent mean ± SD values from three independent experiments. *P < 0.05, **P < 0.01 (n = 4) compared with control group, ^#^ *P* < 0.05, ^##^ *P* < 0.01 compared with mechanical strain or icariin stimulation.

The mRNA expressions of OCN, Col I were investigated using real-time RT-PCR, and the protein expressions of OCN, Col I were investigated by western blot. As shown in Figure 5a, compared to the blank control group, the mRNA expression of OCN was up-regulated significantly (*P* < 0.01) after mechanical strain, combination of mechanical strain stimulation. The combination of mechanical strain and icariin stimulation could up-regulated the mRNA expression of OCN significantly (*P* < 0.01) than that of mechanical strain or icariin alone. The protein expression of OCN was up-regulated significantly (*P* < 0.01) after mechanical strain, icariin, combination of mechanical strain and icariin stimulation compared to blank control group (Figure 5b). The combination of mechanical strain and icariin stimulation could up-regulated the protein expression of OCN significantly (*P* < 0.01) compared to the stimulation of mechanical strain or icariin alone.

**Figure 6:**
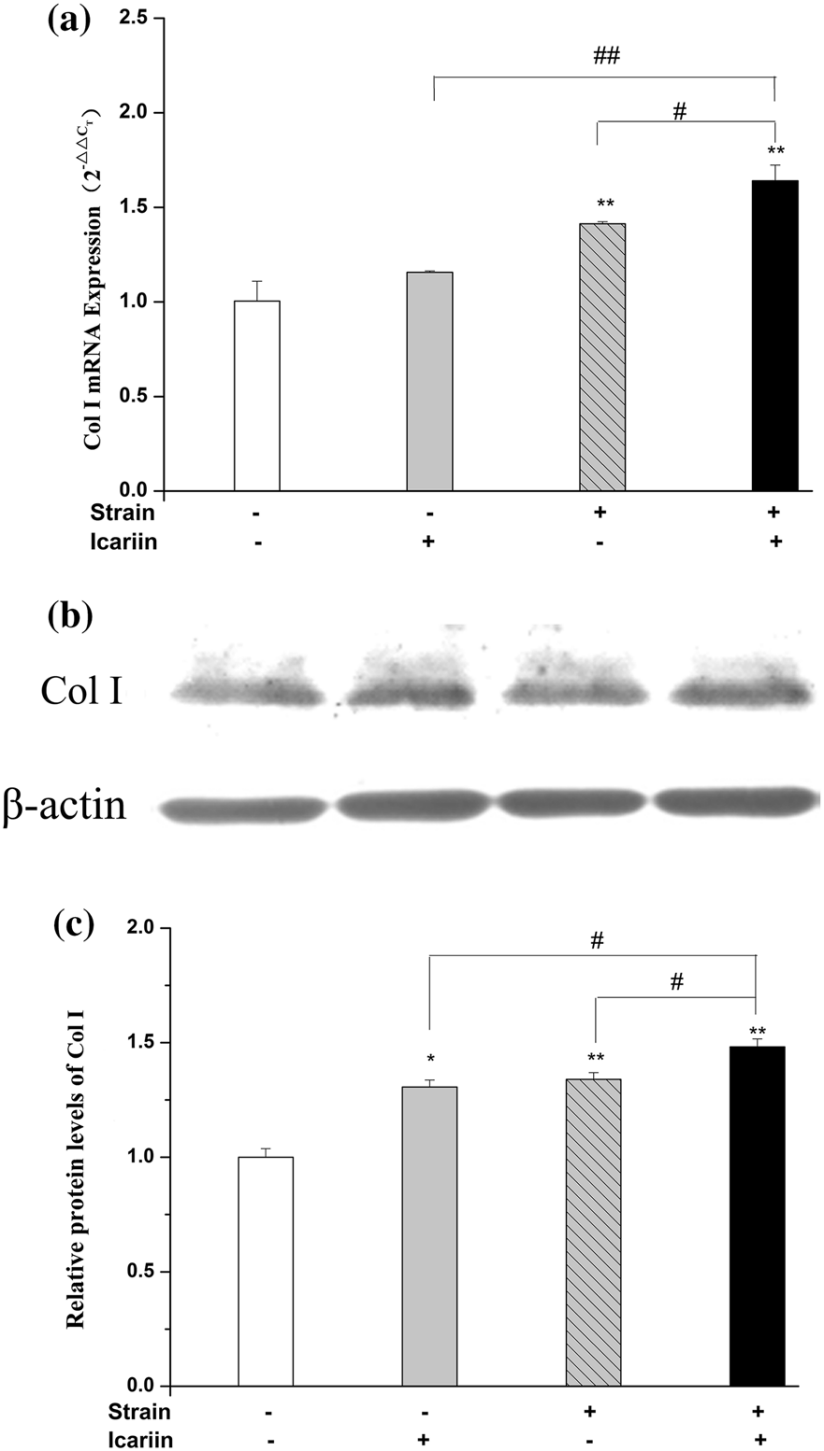
Effect of mechanical strain, icariin, the combination of mechanical strain and icariin stimulation on the protein and mRNA expression of Col I. After stimulation of mechanical strain, icariin, the combination of mechanical strain and icariin on MC3T3-E1 cells of 1 h/day for 3 days, the total protein and mRNA of MC3T3-E1 cells were extracted. The mRNA expression of Col I was analyzed by real-time RT-PCR (**a**), and the protein expression of Col I (**b**) was detected by western blot. The β-actin protein was used as internal control. Optical density analysis (**c**) was obtained with the Image-Pro Plus version 6.0. Data represent mean ± SD values from three independent experiments. *P < 0.05, **P < 0.01 (n = 4) compared with control group, ^#^ *P* < 0.05, ^##^ *P* < 0.01 compared with mechanical strain or icariin stimulation.

As shown in Figure 6a, in comparison with the stimulation of mechanical strain or icariin alone, the mRNA expression of Col I was up-regulated significantly (*P* < 0.01 or *P* < 0.05) after the combination of mechanical strain and icariin stimulation. The combination of mechanical strain and icariin stimulation could increase the protein expression of Col I significantly (*P* < 0.05) than that of mechanical strain, icariin alone (Figure 6b).

### Effect of mechanical strain, icariin, combination of mechanical strain and icariin stimulation on regulating NF-κB signaling pathway

**Figure 7:**
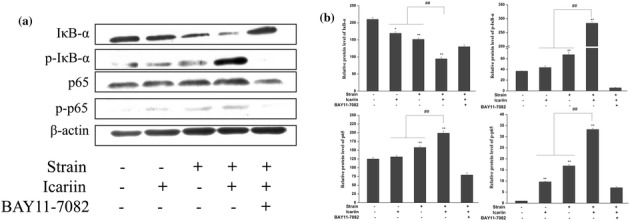
Effect of mechanical strain, icariin, the combination of mechanical strain and icariin stimulation on NF-κB signaling pathway. After stimulation of mechanical strain, icariin, the combination of mechanical strain and icariin on MC3T3-E1 cells of 1 h/day for 3 days, the total protein were then prepared and western blotted for IκB-α, p-IκB-α, p65, P-p65 using specific IκB-α, p-IκB-α, p65, P-p65 antibodies (**a**). The β-actin protein was used as internal control. Optical density analysis (**b**) was obtained with the Image-Pro Plus version 6.0. Data represent mean ± SD values from three independent experiments. *P < 0.05, **P < 0.01 (n = 4) compared with control group, ^#^ *P* < 0.05, ^##^ *P* < 0.01 compared with mechanical strain or icariin stimulation.

NF-κB signaling is associated with bone metabolism and considered to be an important signal transduction pathway in osteoblasts differentiation [38, 39]. In order to investigate effect of mechanical strain, icariin, combination of mechanical strain and icariin stimulation on NF-κB signaling pathway, we first investigated the expressions of IκB-α, phosphorylation of IκB-α (P-IκB-α), p65, P-p65 (phosphorylation of P-p65) which were the important protein in activation of NF-κB pathway. Figure 7 showed that IκB-α degradation was blocked by mechanical strain, combination of mechanical strain and icariin stimulation significantly (*P* < 0.01 or *P* < 0.05). Furthermore, to determine whether this IκB-α degradation was related to IκB-α phosphorylation, we examined the effect of the mechanical strain, icariin, combination of mechanical strain and icariin stimulation on P-IκB-α by western blot, and found that combination of mechanical strain and icariin stimulation also could increase IκB-α phosphorylation significantly (*P* < 0.01) in comparison with mechanical strain or icariin alone. The combination of mechanical strain and icariin stimulation could increase the expression of p65 and p65 phosphorylation significantly (*P* < 0.01) compared to mechanical strain or icariin alone. NF-κB inhibitor BAY 11-7082 was used to investigate the activation of NF-κB pathway, BAY 11-7082 could inhibit the activation of NF-κB by the combination of mechanical strain and icariin stimulation. The β-actin protein was used as internal controls.

## Discussion

Mechanical loading is considered as a fundamental physiological factor for regulating the structure and function of bones, moreover, absence of mechanical loading could lead to the loss of bone mass, while suitable mechanical force could stimulate bone formation [40]. Several studies have illustrated that mechanical stresses, such as compression or tension, can elicit cellular responses [41]. Osteoblasts, deriving from the MSCs, locate on the surface of cancellous bone and cortical bone. Osteoblasts, which are the crucial effector cells sensitive to mechanical stimulation, could be activated to promote bone formation. Our previous studies have shown that mechanical strain could induce osteoblast proliferation, differentiation and mineralization in mouse osteoblast-like cells (MC3T3-E1) [13, 32, 42]. In this study we compared the effect of combination of mechanical strain and drugs (icariin) stimulation to mechanical strain stimulation or drugs alone on inducing osteoblast proliferation, differentiation, and the possible mechanism was investigated in mouse osteoblast-like cells (MC3T3-E1).

In present study, we have introduced a whole cell ELISA that detected the BrdU incorporation during DNA synthesis to evaluate the proliferation of MC3T3-E1 cells after stimulation of mechanical strain, icariin, combined of mechanical strain and icariin in vitro. The cell proliferation with combination of mechanical strain and icariin stimulation was significantly higher than that with the stimulation of mechanical strain or icariin (*P* < 0.01), which revealed that the combination of mechanical load and drugs could promote the cell proliferation of osteoblast significantly compared to stimulation of mechanical load or drugs alone.

ALP, Col I and OCN are the major biological markers in osteoblasts differentiation. Our study showed that ALP activity and mRNA expression were increased significantly by the combination of mechanical strain and icariin stimulation compared to stimulation of mechanical strain or icariin alone. Moreover, the mRNA and protein expression of Col I and OCN were up-regulated remarkably by the combination of mechanical strain and icariin stimulation than that of mechanical strain or icariin alone. The results of protein expression corresponded with the results of mRNA expression on ALP, Col I and OCN, which suggested that the combination of mechanical strain and icariin stimulation on protein expression might have resulted from the transcriptional increasing of ALP, Col I and OCN gene. The results showed that there was a coupling effect of mechanical strain and icariin on the mRNA and protein expression of ALP, Col I and OCN, which suggested that combination of mechanical strain and drug (icariin) stimulation would induce osteoblast proliferation, differentiation better compared to mechanical single strain or drug (icariin) stimulation.

BMPs, potent osteoblastic differentiation factors, play an important role in the regulatory signaling network of bone formation and remodeling [43]. BMP-2 and BMP-4, the vital members of BMPs families, have the ability to promote chondrogenesis, osteogenesis, and have been paid more attention to the field of biomechanics [44]. The present study showed that mechanical strain, icariin, combination of mechanical strain and icariin stimulation could up-regulate the mRNA of BMP-2 and BMP-4 significantly compared to control group, and the protein expression of BMP-2 was also enhanced significantly. Moreover, compared to mechanical strain or icariin stimulation, the combination of mechanical strain and icariin stimulation made a significant increasing of BMP-2 mRNA and protein expression.

NF-κB signaling was associated with bone metabolism and considered to be the important signal transduction pathway in osteoblasts differentiation [45]. In resting cells, the NF-κB heterodimer was held in the cytoplasm through interaction with IκB inhibitory proteins [46]. NF-κB activation resulted from the phosphorylation and proteasome-mediated degradation of inhibitory IκB proteins, and the phosphorylation of IκB proteins was the key point in the regulation of NF-κB pathway in mechanical responses, which was followed by the nuclear translocation and DNA binding of NF-κB, where it could activate the target genes [47]. The present study showed that the combination of mechanical strain and icariin stimulation could increase the degradation and P-IκB-α significantly (P < 0.01) compared to mechanical strain or icariin stimulation. Moreover, p65 protein, one of transcription factors in the NF-κB/Rel family, contained a C-terminal activation domain that was crucial to induce target genes expression in NF-κB pathway. In present study, the phosphorylation of p65 was increased significantly (*P* < 0.01) by the combination of mechanical strain and icariin stimulation compared to mechanical strain or icariin stimulation. The combination of mechanical strain and icariin stimulation could up-regulate BMP-2/BMP-4 expression by activating the NF-κB pathway to enhance osteogenic gene expressions.

## Conclusions

Although the mechanical strain promoted the proliferation, differentiation of osteoblast-like cells (MC3T3-E1) reported by our previous studies, the combination of mechanical strain and icariin stimulation on it was first explored. The present study demonstrated that there was a coupling effect of mechanical strain and icariin, which could not only significantly increase the expression of ALP, Col I, OCN, but up-regulate the expression of BMP-2/BMP-4 by the increase of signaling cascades leading to the activation of NF-κB compared to single mechanical strain or icariin stimulation on the proliferation, differentiation of osteoblast-like cells. The present study will provide scientific evidence that combination of exercise and drug could better than single exercise recovery or drug on treatment of bone injury and remodeling in clinic.

## Abbreviations

ALP: alkaline phosphatase
OCN: osteocalcin
Col I: type I collagen
BMP-2: bone morphogenetic protein-2
NF-κB: nuclear transcription factor kappa-B
IκBα: inhibitor of κBα
P-IκB-α: phosphorylation of IκB-α
ECM: extracellular matrix
BrdU: 5-bromo-20-deoxyuridine
PVDF: polyvinylidene difluoride
TTBS: Tween 20/Tris-buffered saline
Cell ELISA: cell enzyme-linked immunosorbent assay.

## References

[1] Hillam RA, Skerry T (1995). Inhibition of bone resorption and stimulation of formation by mechanical loading of the modeling rat ulna in vivo. J Bone Miner Res.

[2] Hughes-Fulford M (2004). Signal transduction and mechanical stress. Sci STKE..

[3] Duquette TL, Watson DJ (2010). Femoral neck stress fracture in a military trainee. J Orthop Sports Phys Ther.

[4] Burr DB, Milgrom C, Fyhrie D, Forwood M, Nyska M, Finestone A (1996). In vivo measurement of human tibial strains during vigorous activity. Bone.

[5] Frost HM (1992). Perspectives: bone’s mechanical usage windows. Bone Miner.

[6] Mullender M, El Haj AJ, Yang Y, van Duin MA, Burger EH, Klein-Nulend J (2004). Mechanotransduction of bone cells in vitro: mechanobiology of bone tissue. Med Biol Eng Comput.

[7] Schriefer JL, Warden SJ, Saxon LK, Robling AG, Turner CH (2005). Cellular accommodation and the response of bone to mechanical loading. J Biomech.

[8] Rubin J, Rubin C, Jacobs CR (2006). Molecular pathways mediating mechanical signaling in bone. Gene.

[9] Wozniak M, Fausto A, Carron CP, Meyer DM, Hruska KA (2000). Mechanically strained cells of the osteoblast lineage organize their extracellular matrix through unique sites of alphavbeta3-integrin expression. J Bone Miner Res.

[10] Simmons CA, Matlis S, Thornton AJ, Chen S, Wang CY, Mooney DJ (2003). Cyclic strain enhances matrix mineralization by adult human mesenchymal stem cells via the extracellular signal-regulated kinase (ERK1/2) signaling pathway. J Biomech.

[11] Bhatt KA, Chang EI, Warren SM, Lin SE, Bastidas N, Ghali S (2007). Uniaxial mechanical strain: an in vitro correlate to distraction osteogenesis. J Surg Res.

[12] Canalis E, Economides AN, Gazzerro E (2003). Bone morphogenetic proteins, their antagonists, and the skeleton. Endocr Rev.

[13] Wang L, Li JY, Zhang XZ, Liu L, Wan ZM, Li RX (2012). Involvement of p38MAPK/NF-kappaB signaling pathways in osteoblasts differentiation in response to mechanical stretch. Ann Biomed Eng.

[14] Ehrlich PJ, Lanyon LE (2002). Mechanical strain and bone cell function: a review. Osteoporos Int.

[15] Martin TJ, Gaddy D (2006). Bone loss goes beyond estrogen. Nat Med.

[16] Wang C, Zhang C, Han J, Wu H, Fan Y (2011). Simulated evolution of the vertebral body based on basic multicellular unit activities. J Bone Miner Metab.

[17] Perrien DS, Achenbach SJ, Bledsoe SE, Walser B, Suva LJ, Khosla S (2006). Bone turnover across the menopause transition: correlations with inhibins and follicle-stimulating hormone. J Clin Endocrinol Metab.

[18] Liang F, Gardner DG (1999). Mechanical strain activates BNP gene transcription through a p38/NF-kappaB-dependent mechanism. J Clin Invest.

[19] Kumar A, Murphy R, Robinson P, Wei L, Boriek AM (2004). Cyclic mechanical strain inhibits skeletal myogenesis through activation of focal adhesion kinase, Rac-1 GTPase, and NF-kappaB transcription factor. FASEB J.

[20] Granet C, Boutahar N, Vico L, Alexandre C, Lafage-Proust MH (2001). MAPK and SRC-kinases control EGR-1 and NF-kappa B inductions by changes in mechanical environment in osteoblasts. Biochem Biophys Res Commun.

[21] Baldwin AS (1996). The NF-kappa B and I kappa B proteins: new discoveries and insights. Annu Rev Immunol.

[22] Baeuerle PA, Baltimore D (1996). NF-kappa B: ten years after. Cell.

[23] Surh YJ, Chun KS, Cha HH, Han SS, Keum YS, Park KK (2001). Molecular mechanisms underlying chemopreventive activities of anti-inflammatory phytochemicals: down-regulation of COX-2 and iNOS through suppression of NF-kappa B activation. Mutat Res.

[24] Lappas M, Permezel M, Georgiou HM, Rice GE (2002). Nuclear factor kappa B regulation of proinflammatory cytokines in human gestational tissues in vitro. Biol Reprod.

[25] Hsieh CP, Chiou YL, Lin CY (2010). Hyperbaric oxygen-stimulated proliferation and growth of osteoblasts may be mediated through the FGF-2/MEK/ERK 1/2/NF-kappaB and PKC/JNK pathways. Connect Tissue Res.

[26] Nian H, Ma MH, Nian SS, Xu LL (2009). Antiosteoporotic activity of icariin in ovariectomized rats. Phytomedicine.

[27] Mok SK, Chen WF, Lai WP, Leung PC, Wang XL, Yao XS (2010). Icariin protects against bone loss induced by oestrogen deficiency and activates oestrogen receptor-dependent osteoblastic functions in UMR 106 cells. Br J Pharmacol.

[28] Song L, Zhao J, Zhang X, Li H, Zhou Y (2013). Icariin induces osteoblast proliferation, differentiation and mineralization through estrogen receptor-mediated ERK and JNK signal activation. Eur J Pharmacol.

[29] NASA. Strong bones and fewer renal stones for astronauts. International Space Station Program Science Office, NASA’s Johnson Space Center. 2012. http://www.nasa.gov/mission_pages/station/research/news/Strong_Bones.html. Accessed 10 May 2015.

[30] Tang LL, Wang YL, Pan J, Pan J, Cai SX (2004). The effect of step-wise increased stretching on rat calvarial osteoblast collagen production. J Biomech.

[31] Wang L, Zhang X, Guo Y, Chen X, Li R, Liu L (2010). Involvement of BMPs/Smad signaling pathway in mechanical response in osteoblasts. Cell Physiol Biochem.

[32] Yan YX, Gong YW, Guo Y, Lv Q, Guo C, Zhuang Y (2012). Mechanical strain regulates osteoblast proliferation through integrin-mediated ERK activation. PLoS One.

[33] Bottlang M, Simnacher M, Schmitt H, Brand RA, Claes L (1997). A cell strain system for small homogeneous strain applications. Biomed Tech (Berl).

[34] Owan I, Burr DB, Turner CH, Qiu J, Tu Y, Onyia JE (1997). Mechanotransduction in bone: osteoblasts are more responsive to fluid forces than mechanical strain. Am J Physiol.

[35] Cui YL, Qi AD, Liu WG, Wang XH, Wang H, Ma DM (2003). Biomimetic surface modification of poly(l-lactic acid) with chitosan and its effects on articular chondrocytes in vitro. Biomaterials.

[36] Wang QS, Xiang Y, Cui YL, Lin KM, Zhang XF (2012). Dietary blue pigments derived from genipin, attenuate inflammation by inhibiting LPS-induced iNOS and COX-2 expression via the NF-kappaB inactivation. PLoS One.

[37] Wang QS, Cui YL, Dong TJ, Zhang XF, Lin KM (2012). Ethanol extract from a Chinese herbal formula, “Zuojin Pill”, inhibit the expression of inflammatory mediators in lipopolysaccharide-stimulated RAW 264.7 mouse macrophages. J Ethnopharmacol.

[38] Hess K, Ushmorov A, Fiedler J, Brenner RE, Wirth T (2009). TNFalpha promotes osteogenic differentiation of human mesenchymal stem cells by triggering the NF-kappaB signaling pathway. Bone.

[39] Yoshitake F, Itoh S, Narita H, Ishihara K, Ebisu S (2008). Interleukin-6 directly inhibits osteoclast differentiation by suppressing receptor activator of NF-kappaB signaling pathways. J Biol Chem.

[40] Rubin CT, Lanyon LE (1984). Regulation of bone formation by applied dynamic loads. J Bone Joint Surg Am.

[41] Barbee KA, Macarak EJ, Thibault LE (1994). Strain measurements in cultured vascular smooth muscle cells subjected to mechanical deformation. Ann Biomed Eng.

[42] Guo Y, Zhang CQ, Zeng QC, Li RX, Liu L, Hao QX (2012). Mechanical strain promotes osteoblast ECM formation and improves its osteoinductive potential. Biomed Eng Online.

[43] Urist MR (1972). Osteoinduction in undemineralized bone implants modified by chemical inhibitors of endogenous matrix enzymes. A preliminary report. Clin Orthop Relat Res.

[44] Miyazono K, Maeda S, Imamura T (2005). BMP receptor signaling: transcriptional targets, regulation of signals, and signaling cross-talk. Cytokine Growth Factor Rev.

[45] Ryu B, Qian ZJ, Kim SK (2010). Purification of a peptide from seahorse, that inhibits TPA-induced MMP, iNOS and COX-2 expression through MAPK and NF-kappaB activation, and induces human osteoblastic and chondrocytic differentiation. Chem Biol Interact.

[46] Baeuerle PA, Henkel T (1994). Function and activation of NF-kappa B in the immune system. Annu Rev Immunol.

[47] Brown K, Park S, Kanno T, Franzoso G, Siebenlist U (1993). Mutual regulation of the transcriptional activator NF-kappa B and its inhibitor, I kappa B-alpha. Proc Natl Acad Sci USA.

